# Nitrated α-Synuclein Induces the Loss of Dopaminergic Neurons in the Substantia Nigra of Rats

**DOI:** 10.1371/journal.pone.0009956

**Published:** 2010-04-08

**Authors:** Zhongwang Yu, Xiaohui Xu, Zhenghua Xiang, Jianfeng Zhou, Zhaohuan Zhang, Chun Hu, Cheng He

**Affiliations:** Institute of Neuroscience and Key Laboratory of Molecular Neurobiology of Minister of Education, Neuroscience Research Center of Changzheng Hospital, Second Military Medical University, Shanghai, China; National Institutes of Health, United States of America

## Abstract

**Background:**

The pathology of Parkinson's disease (PD) is characterized by the degeneration of the nigrostriatal dopaminergic pathway, as well as the formation of intraneuronal inclusions known as Lewy bodies and Lewy neurites in the substantia nigra. Accumulations of nitrated α-synuclein are demonstrated in the signature inclusions of Parkinson's disease. However, whether the nitration of α-synuclein is relevant to the pathogenesis of PD is unknown.

**Methodology/Principal Findings:**

In this study, effect of nitrated α-synuclein to dopaminergic (DA) neurons was determined by delivering nitrated recombinant TAT-α-synuclein intracellular. We provide evidence to show that the nitrated α-synuclein was toxic to cultured dopaminergic SHSY-5Y neurons and primary mesencephalic DA neurons to a much greater degree than unnitrated α-synuclein. Moreover, we show that administration of nitrated α-synuclein to the substantia nigra pars compacta of rats caused severe reductions in the number of DA neurons therein, and led to the down-regulation of D_2_R in the striatum *in vivo*. Furthermore, when administered to the substantia nigra of rats, nitrated α-synuclein caused PD-like motor dysfunctions, such as reduced locomotion and motor asymmetry, however unmodified α-synuclein had significantly less severe behavioral effects.

**Conclusions/Significance:**

Our results provide evidence that α-synuclein, principally in its nitrated form, induce DA neuron death and may be a major factor in the etiology of PD.

## Introduction

Parkinson's disease (PD) is characterized by the progressive degeneration of the nigrostriatal dopaminergic pathway, as well as the formation of neuronal inclusions known as Lewy bodies and Lewy neurites within DA neurons of the substantia nigra [1]. Although most cases of PD are sporadic, a number of genes have been linked to familial forms of PD. Among these, the α-synuclein gene has received much attention. Missense mutations in α-synuclein and duplications or triplications of the locus cause PD [2]–[7]. α-Synuclein is involved in dopamine neurotransmission, including release and reuptake [8], [9], and also affects enzymes involved in chromatin remodeling [10]. α-Synuclein comprises a major component of Lewy bodies and Lewy neurites in DA neurons in the substantia nigra of patients with PD, and is a major constituent of other types of inclusions found in a group of diseases, collectively known as ‘synucleinopathies’ [11]. Overexpression of α-synuclein in the nigrostriatal system induces PD-like neurodegeneration [12]. The production of α-synuclein in transgenic mice [13], flies [14] or primates [15] leads to motor deficits and neuronal inclusions reminiscent of PD.

Reactive oxygen and nitrogen species are thought to be pivotal to DAergic-specific neurotoxic processes in PD [16], [17]. Peroxynitrite (ONOO^−^), a stable reactive nitrogen species, is a potent oxidant formed by the reaction of nitric oxide with superoxide anions and has been shown to selectively nitrate protein tyrosine residues [18]. Protein nitration and consequent alterations in function are implicated as important contributors to cell dysfunction and apoptosis in neurodegenerative pathologies [19]. By exposing DAergic cells to pan-nitrating reagents, certain nitrated proteins have been detected in DA neurons, including protein kinase C [20], catecholamines [21], manganese superoxide dismutase [22], α-synuclein [23]–[25], which demonstrated the possible importance of nitrated stress in the etiology of PD both *in vitro* and *in vivo*. However, it remains unclear which specific nitrated proteins are toxic to DAergic cells, and contribute to the development of PD.

Previous reports have shown that nitrated α-synuclein was detected in brain tissue from individuals with synucleinopathy, indicating a direct link between oxidative and nitrative damage to the onset and progression of neurodegenerative synucleinopathies [23]. However, the exact role of nitrated α-synuclein in the pathology of PD remains to be elucidated. In the present study, we show that the nitrated form of α-synuclein is significantly more toxic to DA neurons, both *in vitro* and *in vivo*. Furthermore, we show that exposing the substantia nigra to nitrated α-synuclein causes motor defects reminiscent of PD. These results provide compelling insight into the development of synucleinopathies in general, and PD in particular.

## Materials and Methods

### Cloning and preparation of nitrated protein

α-Synuclein cDNA (SNCA112) was generated by PCR from a human brain cDNA library (Clontech, Palo Alto, CA) and inserted into pHA-TAT expression vector (kindly provided by Dr. Steven F Dowdy) that contains a HA-tag in frame with the TAT internalization signal peptide, to generate HA-TAT-α-synuclein (herein refered to as SYN). α-Synuclein lacking the TAT peptide (SYN^TAT−^) was created by amplifying α-synuclein cDNA and cloning it into a pet28a expression vector (Novagen). Full-length eGFP cDNA, amplified from pEGFP-N1 (Clontech, Palo Alto, CA), was subcloned into pHA-TAT expression vector to generate HA-TAT-eGFP (GFP). All constructs were fully sequenced. Recombinant proteins were expressed and purified using standard techniques [26].

Protein nitration was performed as previously reported, with some modifications [27], [28]. Briefly, proteins suspended in PBS [pH 7.4] were incubated with 1 mM 3-morpholinosydnonimine (SIN-1; Sigma-Aldrich, St. Louis, MO, USA) at 37°C for 3 h, then dialyzed in PBS for 24–48 hours with multiple exchanges of PBS. Samples of purified SYN and nitrated-SYN (N-SYN) proteins were fractionated using 15% SDS-PAGE, and stained with Brilliant Coomassie Blue, or detected by immunoblotting with anti-HA antibody (1∶3000, Roche, Indianapolis, IN, USA) or monoclonal mouse anti-Nitrotyrosine antibody (1∶2000, Upstate, Temmecula, CA, USA). After incubation with horseradish peroxidase (HRP)-conjugated secondary antibodies (Sigma; 1∶10,000), protein bands were visualised by chemiluminescence (ECL Western Blotting kit, Amersham). Coomassie Blue-stained protein corresponding to the theoretical molecular weight of SYN (∼20 kD) were excised from gels and stored at −80°C before MALDI-TOF/TOF MS analysis.

### MALDI-TOF/TOF MS analysis

In-gel tryptic digest and mass spectrometry (MS) were performed at the Institute Mass Spectrometry Facility (University of Fudan, Shanghai, China). Briefly, SYN and N-SYN in SDS-PAGE were transferred to 96-well microplates loaded with 100 µl of 50% Acetonitrile (ACN)/25 mM ammonium bicarbonate solution per well. After destaining for 1 h, gel plugs were dehydrated with 100 µl of 100% ACN for 20 min and then dried thoroughly in a SpeedVac concentrator (Thermo Savant, USA) for 30 min. The dried gel particles were rehydrated at 4°C for 45 min with 2 µl/well trypsin (12.5 ng/ml, Promega, Madison, WI, USA) in 25 mM ammonium bicarbonate, and then incubated at 37°C for 12 h. After trypsin digestion, the peptide mixtures were extracted with 100 µl extraction solution (50% ACN/0.1% TFA) per well at 37°C for 1 h. Finally, the extracts were dried under the protection of N_2_.

Peptides were eluted with 0.5 µl matrix solution (α-cyano-4-hydroxy-cinnamic acid (CHCA, Sigma-Aldrich, St. Louis, MO, USA) in 50% ACN/0.1% TFA) before spotting onto target plates. Samples were allowed to air-dry and then analyzed using a 4700 MALDI-TOF/TOF Proteomics Analyzer (Applied Biosystems, CA, USA). Trypsin-digested myoglobin was used to calibrate the mass instrument. All acquired samples spectra were processed using the default mode of 4700 ExploreTM software (Applied Biosystems, CA, USA). Parent mass peaks with a mass range 700–3200 Da and a minimum S/N of 20 were picked out for tandem TOF/TOF analysis. The theoretical molecular mass (m/z) was determined using an ExPASy-computed peptide-mass tool program (http://www.expasy.org/tools/peptide-mass.html), and the following parameters: trypsin digest with one missing cleavage, possible oxidation of methionine (MSO), cysteines in reduced form, peptides with a mass bigger than 500 Da, monoisotopic masses of the occurring amino acid residues, and peptide masses as [M+H]^+^.

### SHSY-5Y and primary ventral mesencephalon cultures and immunocytochemistry

SHSY-5Y DAergic human neuroblastoma cells, kindly provided by Dr. Zhou JW, were cultured at 37°C, 5% CO_2_ and 95% humidity in Dulbecco's Modified Eagles Medium (DMEM) + F12 (1∶1) (Invitrogen, Grand Land, USA) containing 10% fetal bovine serum. Having grown to 80% confluence, cells were transferred onto glass cover slips at a density of 1×10^5^ and kept overnight.

The ventral part of the midbrain was dissected from embryonic day 16 (E16) Sprague-Dawley rats and dissociated in Hanks balanced salt solution (HBSS) containing 0.125% trypsin (GIBCO, Canada) for 20 min at 37°C. Tissues were resuspended in DMEM containing 10% fetal bovine serum (FBS) (BIOSOURCE, Brazil) and 10% horse serum (HS) (GIBCO, Canada), Cells were seeded at 5×10^5^/well onto poly-L-lysine (20 µg/mL) precoated cover slips in 24-well culture plates and maintained at 37°C in a humidified atmosphere of 5% CO_2_ and 95% air, and then switched 12 hours later to serum-free Neurobasal medium (GIBCO, Canada) containing B27 supplement (GIBCO, Canada).

Cells cultured on poly-L-lysine-coated glass coverslips were fixed with 4% paraformaldehyde/PBS solution for 20 min, permeabilized using 0.1% Triton X-100 and 0.2% FBS in PBS for 15 min, and incubated overnight with anti-Tyrosine hydroxylase (TH) antibody (1∶6000, Sigma-Aldrich, St. Louis, MO, USA) in PBS with 1% horse serum at 4°C. After washing with PBS, cells were incubated with FITC- or rhodamine-conjugated secondary antibodies (1∶200, Jackson Immuno Laboratories, West Grove, PA, USA) for 1 h at RT, washed again, and counterstained with Hoechst 33258 (Sigma-Aldrich, St. Louis, MO, USA) for 15 min at RT. Cells were visualized and imaged under an inverted fluorescence microscope (DXM1200, Nikon, Inc., Japan).

To calculate the number of TH-immunoreactive primary neurons on each coverslip, TH-positive cells in at least ten randomly chosen observation fields were counted in each of three independent cultures derived from three individuals for each experimental condition. The data were then expressed as the percentage of TH-positive neurons in SYN, N-SYN or N-GFP treated cultures relative to PBS-treated cultures. At least nine coverslips were counted in each group. Over 3000 DA neurons were counted.

### LDH cytotoxicity assay

Cellular toxicity was evaluated by measuring lactate dehydrogenase (LDH) activity in the medium at each indicated time point after exposure to the CytoTox96 nonradioactive assay (Promega, Madison, WI, USA). LDH activity was then quantified by measuring wavelength absorbance at 490 nm according to the manufacturer's protocol. Data were averaged across three independent experiments, normalized to the amount of LDH released from lysed cells at each time point, and corrected for baseline LDH release from non-treated cells exposed to PBS.

### TUNEL staining

TUNEL staining was performed using the In Situ Cell Death Detection Kit, TMR red (Roche, Mannheim, Germany) according to the manufacturer's instructions. The number of TUNEL-positive was normalized to the total number of Hoechst labeled cells and averaged across three independent experiments, as described previously [29].

### Animal surgery

All animal experiments were carried out in adherence with the National Institutes of Health Guidelines on the Use of Laboratory Animals and approved by the Second Military Medical University Committee on Animal Care. A stainless-steel guide cannula was stereotaxically implanted into the right substantia nigra pars compacta (SNpc) of adult male Sprague-Dawley rats (200–230 g) using the following coordinates: anteroposterior (AP) −4.8 mm; lateral (L), +2.0 mm; and dorsoventral (DV), −7.0 mm, and using the bregma as the starting point. Each rat then received infusions of 2 µl of 20 µM N-SYN, SYN or N-GFP at a rate of 0.5 µl/min everyday for two weeks. Another group of rats were administered a single injection of 2 µl 6-OHDA (10 µg/µl, Sigma-Aldrich, St. Louis, MO, USA) dissolved in 0.02% ascorbate/saline using the same coordinates: anteroposterior (AP) −4.8 mm; lateral (L), +2.0 mm; and dorsoventral (DV), −7.0 mm, and using the bregma as the starting point. This group was included as a positive control.

To evaluate the effects of the treatments, rats were acclimatized for 15 min in a 30 cm diameter, hemispherical pot, and then subcutaneously injected with apomorphine (APO, 0.5 mg/kg in saline; Sigma-Aldrich, St. Louis, MO, USA). Post-injection rotations were counted over 30 min, and visual behavioral assessments were made using video-recorded observations.

### Behavioral tests

Open-field activity was observed in automated activity cages. Rats were adapted daily to the open-field test for one week preceding the injections. Tests were performed at the same time (between 3:30 p.m. and 5:30 p.m.) after 21 days post-surgery. Locomotor activity was recorded by video tracking. The behavior was videotaped for 15 minutes, and the following parameters were recorded: total distance traveled, distance traveled in the center of the field, distance traveled in the periphery, and active time. The scoring was calculated as mean ± SEM. Statistical significance was tested using two-way ANOVA analysis.

The rotorod test was conducted 1 day before and 21 days after stereological surgery, using a standard rotorod apparatus (DigBeha-RRTM, JLsofttech, Shanghai). The parameters of rotorod system include start speed, acceleration and highest speed (3 rpm, accelerate 1 rpm/24 s, 8 rpm). Rats that were unable to stay on the rod for 2 min over 3 trials were excluded from further experimentation. Each rat was placed on the confined section of the rod and was recorded over three consecutive trials with a 2 min rest between each trial. The mean latency to fall for the three trials, measured both as a function of time (sec) and rod velocity (rpm), was used for analysis. In all trials, if the rat did not fall from the rod after 2 min, it was removed.

### Isolation and Processing of Tissues

It was performed according to the previous protocol [30]. Briefly, rats were deeply anaesthetized with sodium pentobarbital (40 mg/kg i.p.). Brains were removed and divided into two parts by a coronal blade cut at approximately −3.5 mm behind bregma. The caudal part containing the SNc was fixed in the ice-cold 4% paraformaldehyde in 0.1 M phosphate buffer (PB), pH 7.4. The rostral piece of brain tissue was used immediately to dissect the right and left striatum. The striatum from each hemisphere was homogenized and separated into two separate tubes. The tissue pieces were weighed, frozen separately on dry ice, and kept at −80°C until assayed for protein expression or dopamine (DA) and 3–4-dihydroxyphenylacetic acid (DOPAC) content. The fixed part of brains were stored overnight at 4°C and then transferred into 20% sucrose in 0.1 M PB for cryoprotection. Coronal sections (20 µm thick) were cut on a freezing stage sliding microtome (CM1900, Leica, Germany) and processed for immunohistochemistry.

### Immunohistochemistry

A series of the total sections throughout the substantia nigra were mounted onto gelatinized slides, incubated with antibody against TH (1∶6000; Sigma-Aldrich, St. Louis, MO, USA), HA (1∶1000; Abcam, Hong Kong, China), GAD (1∶1000; Sigma-Aldrich, St. Louis, MO, USA ), GFAP (1∶200; Sigma-Aldrich, St. Louis, MO, USA), α-synuclein (2E3, 1∶1000; kindly provided by Prof. Chan P) [31] or Iba-1 (1∶500; Abcam, MA, USA) overnight at 4°C, followed by incubation with FITC-conjugated donkey secondary anti-mouse antibody (1∶200; Jacksonimmuno, West Grove, PA, USA), TRITC-conjugated donkey secondary anti-mouse antibody (1∶200; Jacksonimmuno, West Grove, PA, USA), TRITC-conjugated donkey secondary anti-rabbit antibody (1∶200; Jacksonimmuno, West Grove, PA, USA) or TRITC- conjugated donkey secondary anti-goat antibody (1∶200; Jacksonimmuno, West Grove, PA, USA) for 1 h at room temperature. Sections were then counterstained with Hoechst to stain the nuclei. One set of TH immunostaining sections were counterstained with Nissl staining. As a negative control, the primary antibody step was omitted, no staining was observed (data not shown). For double labeling with immunohistochemistry and Thioflavin-T to detect aggregation, sections containing the substantia nigra were incubated with antibody against α-synuclein (2E3, 1∶1000; kindly provided by Prof. Chan P) overnight at 4°C, followed by incubation with TRITC-conjugated donkey secondary anti-mouse antibody (1∶200; Jacksonimmuno, West Grove, PA, USA) for 1 h at room temperature. After subsequent washes, sections were then incubated 8 min with a 0.05% Thioflavin-T solution according to the method described by Albani D [32]. Staining was analyzed and photographed under fluorescence microscopy (DXM1200, Nikon, Inc., Japan) using proper optical filters. As a negative control, the secondary antibody step or the Thioflavin-T step was omitted, and no optical crosstalk was detected (data not shown).

### Cell counting

Cell counting was performed according to the previous protocol with minor modification [33]. Briefly, by applying anti-HA antibody, the anterior and posterior boundaries of the substantia nigra included in the analysis were defined according to the area transduced by HA-TAT-SYN (SYN) in preliminary experiments [approximately anteroposterior (AP) −4.4 mm through −6.0 mm from bregma]. Sections included in the analysis throughout the substantia nigra were analyzed stereologically. Each section was first viewed at low magnification (4×). The third cranial nerve, a line between the medial lemniscus and the tractus opticus basalis, or a line extending dorsally from the most medial boundary of the cerebral peduncle were used as a landmark to define the vertical border between the substantia nigra and the ventral tegmental area; and the medial lemniscus was used as the dorsal border, as described previously [34]. The number of TH-positive cells was counted using higher magnification (20×, 40×). To avoid double counting of neurons with unusual shapes, TH- and Hoechst co-labeled cells were counted only when their nuclei could be distinctly visualized.

### Anti-D2 Western blot

Striatal tissue from treated rats was homogenized on ice in RIPA (50 mM Tris-HCl, pH 7.5, 150 mM NaCl, 0.1% SDS, 1% Triton X-100, 0.5% deoxycholic acid sodium, 1% NP-40, 0.02% NaN_3_) containing a protease inhibitor cocktail (Roche, Germany), followed by centrifugation at 12,000 g for 15 min at 4°C. The supernatant was then separated by 15% SDS-PAGE electrophoresis and immunoblotted with anti-D2 receptor (1∶100; Millipore, USA), anti-TH (1∶8000; Sigma-Aldrich, St. Louis, MO, USA) and anti-GAPDH (1∶10000; KANGCHEN, China) antibodies. Protein bands were visualized by chemiluminescence (ECL Western Blotting kit, Amersham), analysed using Image-Pro Plus software, and expressed as band intensities relative to the GAPDH band in the same lane.

### Determination of Dopamine Content in striatum by HPLC

Striatal levels of dopamine (DA) and 3,4-dihydroxyphenylacetic acid (DOPAC) were measure by HPLC at the Institute of Neuroscience (Chinese Academy of Sciences, Shanghai, China). Briefly, striatal tissues taken from N-SYN, SYN and N-GFP groups 5 weeks post surgery were homogenized in 0.2 M perchloric acid. Insoluble debris was removed by centrifugation at 20,000 rpm for 10 min, and the supernatant was filtered through a Millipore MC cartridge. The filtrate was injected on a C18 reverse phase column (Capcell PAK C18 MgII) and analyzed by HPLC with electrochemical detection (ESA, Bedford, MA). The levels of DA, DOPAC in ipsilateral striatum were expressed as a percentage of that in contralateral striatum.

### Statistical Analysis

All values are expressed as mean ± SEM. Differences among means were analyzed by one-way ANOVA followed by Tukey post hoc test for pairwise comparison unless otherwise stated.

## Results

### Preparation and identification of nitrated α-synuclein

**Figure 1:**
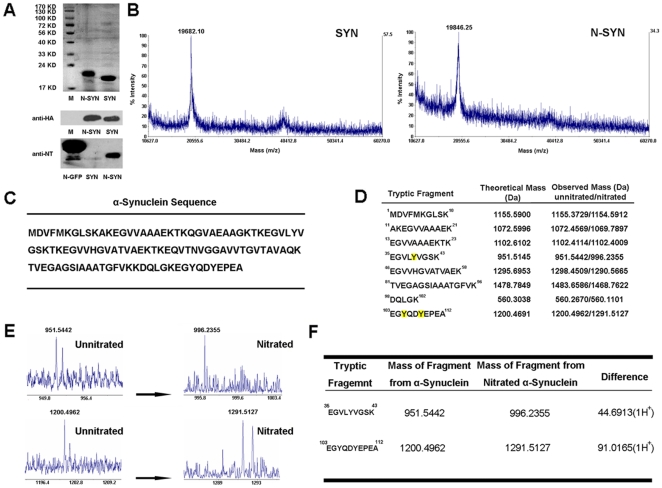
Purification and identification of nitrated α-synuclein. (A) Unnitrated (SYN) and nitrated (N-SYN) α-synuclein TAT-HA fusion proteins were subjected to 15% SDS-PAGE and stained with Coomassie blue (top), and with anti-HA (middle) or anti-NT (N-Tyr) antibody (bottom). Marker (M), nitrated eGFP (N-GFP). (B) Identification SYN and N-SYN by MALDI-TOF-TOF MS. The prominent peak for SYN was 19682.10 m/z (theoretical m/z: 19806, mass accuracy: 0.6%; left-hand panel). The prominent peak for the modified protein, N-SYN, was 19846.25 m/z (theoretical m/z: 19806+45n, where 1≤n≤8; right-hand panel) (C) Amino acid sequence of human α-synuclein (SNCA112). (D) Peptide fragments obtained from MS analysis that match the sequence for unmodified or nitrated α-synuclein. (E) The difference in mass between two major nitrated fragments (right in E) and their corresponding unmodified fragment (left in E) are shown in (F).

Purified α-synuclein fused to TAT-HA (SYN) was exposed to 3-morpholinosydnonimine (SIN-1), a putative nitrating agent that selectively nitrates tyrosine residues [19], [28], [35]. Coomassie Blue R-250 staining revealed that SYN exposed to SIN-1 (N-SYN) separated in SDS-PAGE as a single band with a molecular weight similar to untreated SYN (Figure 1A, top). Anti-HA Western blotting further confirmed that treating SYN with SIN-1 did not change the electrophoretic properties of SYN, and showed that the N-SYN product is slightly larger than SYN (Figure 1A, middle). The nitration of α-synuclein induced by SIN-1 was confirmed with an anti-nitrated tyrosine (NT) Western blot (Figure 1A, bottom).

α-Synuclein has three potential nitrate acceptor tyrosine sites. To determine which residue(s) had been nitrated, SYN and N-SYN bands in SDS-PAGE were solubilized and analyzed by MALDI-TOF/TOF MS. Unmodified SYN (theoretical m/z: 19806) produced a prominent peak (m/z: 19682.10, with a mass accuracy of 0.6%) unique from the prominent peak observed (m/z: 19846.25) for SIN-1-treated SYN (theoretical m/z: 19806+45n, where 1≤n≤8, Figure 1B). The difference in masses between these two peaks, equal to unmodified α-synuclein plus the equivalent mass of three nitrate groups, indicates that all three tyrosine residue acceptor sites in the SYN protein possibly had been nitrated.

Peptides obtained by MS analysis that matched α-synuclein sequence fragments (Figure 1C) are listed in Figure 1D. Among the fragments obtained from nitrated and unmodified SYN (Figure 1D), the mass signal corresponding to residues 103–112 was increased from 1200.4962 m/z to 1291.5127 m/z, and the signal corresponding to residues 35–43 increased from 951.5442 m/z to 996.2355 m/z (Figure 1E). These results indicate that incubating purified SYN with SIN-1 leads to the nitration of both tyrosine residues within the Glu^103^-Ala^112^ fragment (Tyr 105 and Tyr 108), as well as the single tyrosine residue within the Glu^35^-Lys^43^ fragment (Tyr 39; Figure 1F).

### Nitrated α-synuclein is sufficient to induce neurotoxicity *in vitro*

**Figure 2:**
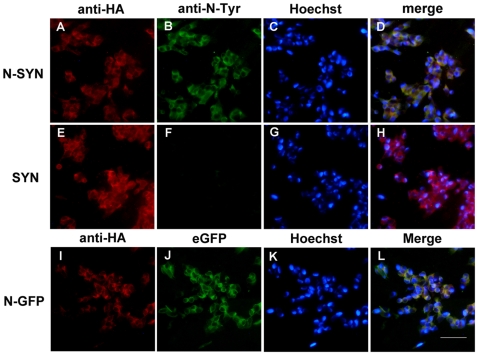
TAT-mediated internalization of fusion proteins into SHSY-5Y cells. SHSY-5Y cells were grown under standard culture conditions, and then incubated with 0.1 µM of N-SYN (A–D), SYN (E–H) or N-GFP (I–L) for 20 min. All three HA-tagged (red; A, E and I) fusion proteins localized to the cytoplasm. Nitration of N-SYN or SYN were determined by fluorescent labeling of anti-N-Tyr antibody (green; B and F). N-GFP proteins were visualized by excitation of eGFP (green; J). The nuclei were stained with Hoechst (blue; C, G and K). Merged images are shown in (D), (H) and (L). Scale bar, 50 µm.

**Figure 3:**
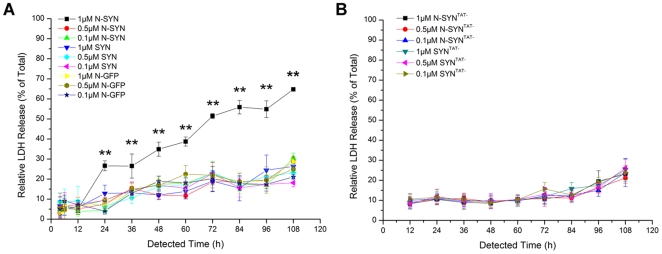
Intracellular but not extracellular N-SYN is detrimental to SHSY-5Y cells in a dose-dependent manner. (A) Graph showing relative LDH release in response to treatment with various concentrations of the three fusion proteins. SHSY-5Y cells were incubated with the indicated concentration of N-SYN, SYN or N-GFP. At each indicated incubation time point, equal aliquots of supernatant were collected and assayed for LDH release. (B) SHSY-5Y cells were incubated with the indicated concentration of N-SYN^TAT−^ or SYN^TAT−^, fusion proteins that lack the TAT peptide. N-SYN^TAT−^ and SYN^TAT−^ showed no apparent cytotoxicity to SHSY-5Y cells, as measured by LDH release. Data represent mean ± SEM from at least three independent experiments. **, *p*<0.01.

To evaluate the survival of DA neurons exposed to SYN or N-SYN, we incubated human DAergic SHSY-5Y cells with different concentrations (0.1 µM–1 µM) of nitrated or unnitrated TAT-fused SYN. The intracellular localization of N-SYN in SHSY-5Y cells was confirmed by anti-HA immunocytochemistry. Consistent with previous reports [36], the TAT motif facilitated the internalization of SYN into the cytoplasm of almost all cultured cells. N-SYN was also effectively directed to the intracellular space of SHSY-5Y cells, indicating that nitrating does not affect internalization and demonstrating that all three fusion proteins, including SYN, N-SYN, and a nitrated form of eGFP also fused to TAT-HA (N-GFP); can localize to the cytoplasm (Figure 2). Cytotoxicity was then determined using a lactate dehydrogenase (LDH) release assay. At low concentrations (from 0.1 to 0.5 µM), neither SYN nor N-SYN caused increases in LDH release from SHSY-5Y cells relative to N-GFP, at any time point up to 108 h of observation. However, within 24 h of applying 1 µM N-SYN, but not SYN, there was a significant increase in LDH release that continued to rise over time relative to N-GFP control and SYN levels, indicating a dramatic cytotoxic effect (Figure 3A).

To determine whether cytotoxicity was dependent on the uptake of N-SYN into the cell, we exposed SHSY-5Y to nitrated or unnitrated SYN fusion proteins lacking the TAT peptide, SYN^TAT-^. Removing the TAT peptide completely eliminated the increase in LDH release, showing that intracellular, but not extracellular, N-SYN is deleterious to cell survival (Figure 3B). This finding also shows that the observed cytotoxicity was not due to secondary reactive nitrate species within the N-SYN solution, such as peroxynitrite, since these are able to cross cell membranes [37].

### N-SYN induces DAergic cell apoptosis in a dose-dependent manner

**Figure 4:**
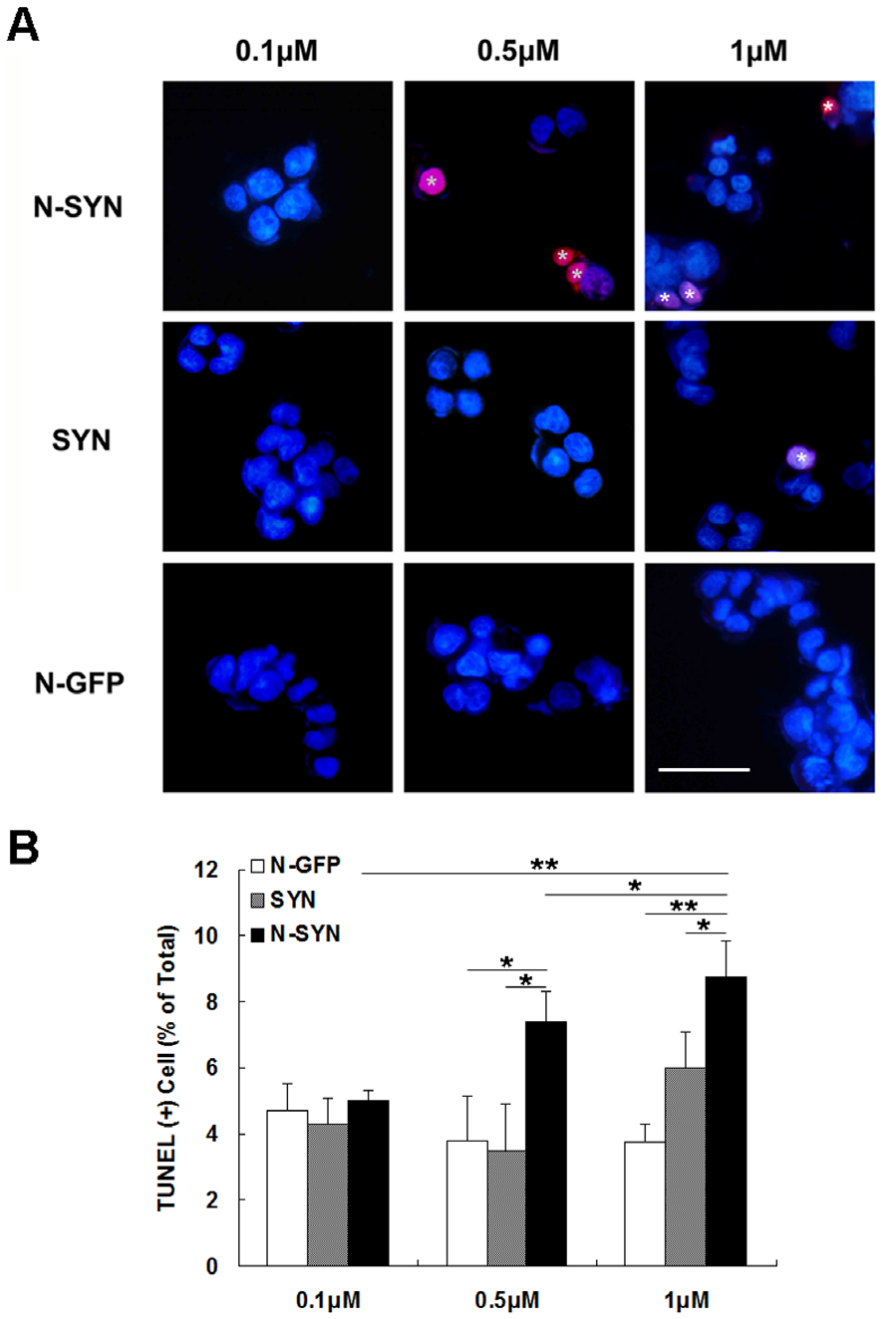
N-SYN induces apoptosis of SHSY-5Y cells. Apoptosis was determined by TUNEL staining after incubating SHSY-5Y cells with the indicated concentration of N-SYN, SYN or N-GFP for 5 h. Representative images of cells in each treatment group are shown in (A). Typical apoptotic cells (marked by asterisks) displayed chromatin condensation accompanied by shrinking of the soma (Hoechst, blue; TUNEL, red). (B) Quantification of the proportion of apoptotic cells in each treatment group. Data are expressed as percent of TUNEL-positive *vs*. Hoechst-positive cells and presented as mean ± SEM from at least three independent experiments. *, *p*<0.05; **, *p*<0.01. Scale bar, 40 µm.

SHSY-5Y cells exposed to SYN and N-SYN were TUNEL labeled to assess cellular apoptosis (Figure 4). Apoptotic cells were apparent 5 hours after application of 0.5 µM of N-SYN. 1 µM of N-SYN had a more dramatic effect, causing a more than 2-fold increase in the percentage of apoptotic SHSY-5Y neurons in the culture dish compared to N-GFP treated control neurons. Although 1 µM SYN also increased the incidence of apoptotic cell death, N-SYN was significantly more toxic at this concentration. Thus, nitrated α-synuclein is more toxic to human DAergic SHSY-5Y cells than the unmodified form of the protein. To determine if there was any correlation between protein aggregation and apoptotic cell death of SHSY-5Y cells, anti-HA antibody was used to detect protein aggregation. Immunostaining with HA and TUNEL revealed that some TUNEL positive apoptotic cells contained intracellular aggregates (Figure S1, arrow), but some apoptotic cells didn't contain intracellular aggregates (Figure S1, arrow head). Additionally, some cells contained HA positive aggregates with TUNEL negative (Figure S1, asterisk). Thus, correlation between protein aggregation and apoptotic cell death still need clarified.

### Nitrated α-synuclein induces the death of primary ventral mensencephalic DA neurons

**Figure 5:**
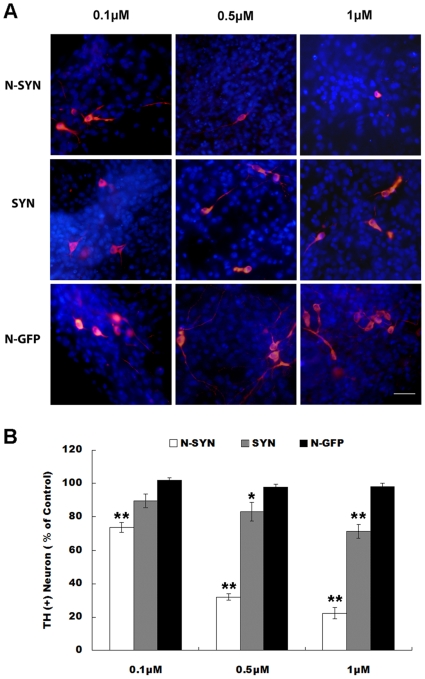
DAergic neurotoxicity induced by N-SYN. Primary cultures of mesencephalic cells incubated with the indicated concentration of N-SYN, SYN or N-GFP for 48h were stained with anti-TH antibody, followed by Hoechst staining. (A) Representative images of cells from each treatment group. (B) Quantification of the proportion of TH-positive DA neurons in each group. Data represent mean ± SEM from three independent experiments. *, *p*<0.05; **, *p*<0.01. Red, TH; blue, Hoechst. Scale bar, 40 µm.

To further assess neurotoxicity, dissociated primary ventral mensencephalic (VM) cells from the brains of E16 rats were exposed to N-SYN for 48 h, and TH-positive DA neurons were counted. High concentrations of N-SYN (5 and 10 µM) were found to be highly toxic, killing most cells, including glia (data not shown). Lower concentrations, on the other hand, greatly reduced the number of DA neurons in a dose-dependent manner (Figure 5). Incubation with 0.1, 0.5 and 1 µM N-SYN reduced DA neurons to 73.6±3.0%, 32.0±2.0% and 22.2±3.3%, respectively, compared to control cultures treated with N-GFP (*p*<0.01). As expected from previously published [32], 0.5 µM and 1 µM SYN also reduced the number of DA neurons, however to a significantly lesser degree than N-SYN (89.6±4.1%, 83.1±5.5% and 71.6±4.1% of control levels). These results demonstrate that nitrated α-synuclein is more toxic to cultured ventral mensencephalic DA neurons than unmodified α-synuclein.

### N-SYN leads to the death of DA neurons in the substantia nigra pars compacta

**Figure 6:**
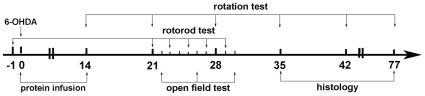
Schematic time line of the experimental design. Stereotaxically administrations of N-SYN, SYN and N-GFP into the right substantia nigra were processed from day 0 to 14, and single injection of 6-OHDA was processed on day 0. The open field test was conducted after 21 days post-surgery. The rotorod test was conducted 1 day before and 21 days after stereological surgery. The rotation test was conducted on day 14, 21, 28, 35, 42, 77. Histology was processed on 5 weeks and 11 weeks postsurgery.

**Figure 7:**
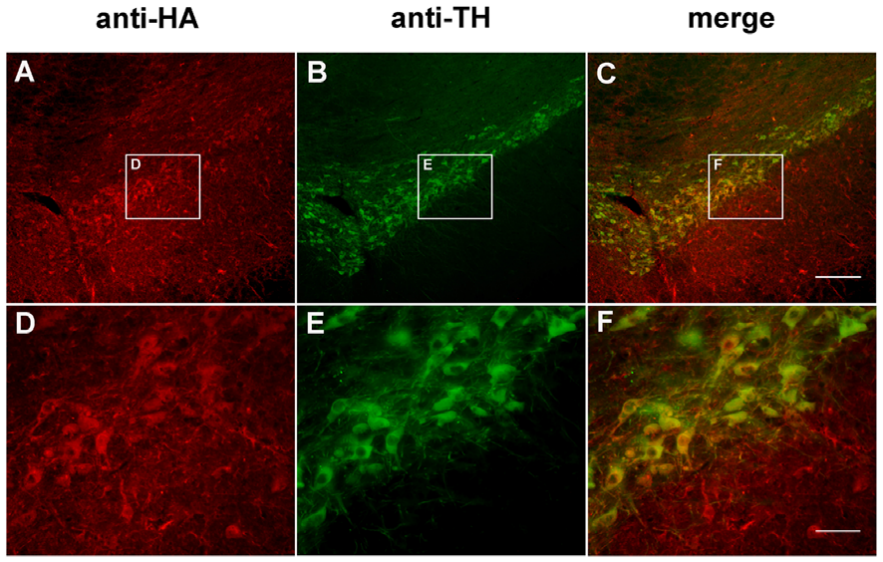
TAT-mediated internalization of fusion proteins into substantia nigra of rats. N-SYN was stereotactically injected in the substantia nigra of rats. After 24 hours, the nigral DA neurons were specifically labeled with anti-HA Ab (A, D) and anti-TH Ab (B, E). Detection with anti-HA Ab revealed the fusion protein localized in almost all cells in SNpc, including DA neurons (C, F, merge) in the injected hemisphere. No anti-HA staining was observed on the contralateral side (data not shown). SYN also localized in almost all cells in SNpc (data not shown). Scale bars: (A–C), 200 µm; (D-F), 50 µm.

**Figure 8:**
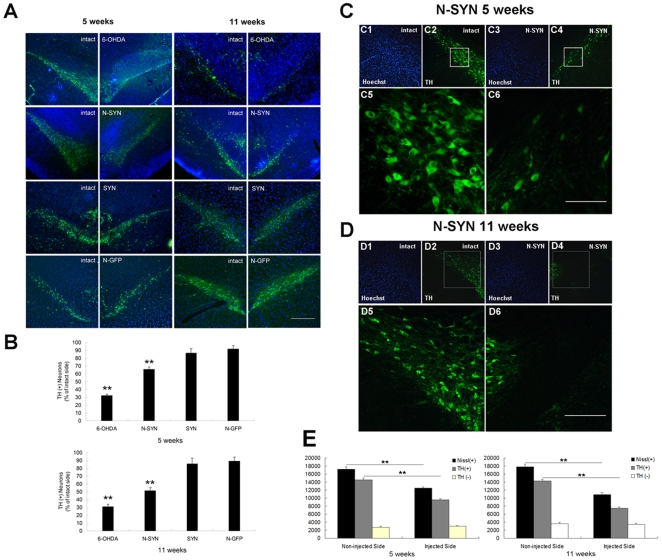
Ipsilateral DA neuron lesions in the SNpc after N-SYN infusion. (A) Representative rat coronal sections showing populations of TH-positive DA neurons in the SNpc 5 weeks and 11 weeks after ipsilateral infusion of 6-OHDA, N-SYN, SYN or N-GFP. Compared to the 6-OHDA as well as to the N-SYN groups, DA neurons in the SYN and N-GFP groups were relatively spared. TH, green; Hoechst, blue. TH-positive DA neurons in the SNpc were more vulnerable to N-SYN than SYN or N-GFP treatment (A, B). (B) Histograms showing the percentage of TH-positive DA neurons on the injected side relative to that on the intact side 5 weeks and 11 weeks after rats were ipsilaterally infused with N-SYN, SYN, 6-OHDA or N-GFP. The percent of TH-positive neurons from both infused and control sides of the SNpc were severely reduced in the 6-OHDA treated group (32.4±1.5% at 5 weeks; 30.7±3.0% at 11 weeks) and the N-SYN treated group (65.5±3.0% at 5 weeks; 51.3±4.0% at 11 weeks), but were mildly reduced in the SYN-treated group (86.6±5.4% at 5 weeks; 85.5±7.7% at 11 weeks). (C) and (D) Representative rat coronal sections showing a substantially reduced population of DA neurons in the SNpc 5 (C) and 11 (D) weeks after ipsilateral infusion of N-SYN. C5, C6, D5 and D6 show high magnification views of the boxed area in C2, C4, D2 and D4, respectively. Hoechst staining (blue; C1, C3, D1 and D3) was conducted to rule out the possibility that the reduction in TH-positive DA neurons was due to a reduction in the total number of cells in the detection area. (E) Further quantification of Nissl-positive and TH-positive neurons on both ipsi- and contralateral sides of SNpc in N-SYN group. Scale bars: (A), 0.5 mm; (C), 50 µm; (D), 100 µm. Values represent mean ± SEM; **, *p*<0.01.

To investigate the effect of N-SYN *in vivo*, we administered N-SYN, SYN or 6-OHDA (a neurotoxin that selectively kills DA neurons) into the ipsilateral substantia nigra pars compacta (SNpc) of adult male rats and compared the number of DA neurons therein with that in the contralateral SNpc after 5 or 11 weeks (Figure 6). The intracellular localization of N-SYN in DA neurons was confirmed by anti-HA immunohistochemistry. Consistent with previous reports [36], the TAT motif facilitated the internalization of SYN into the cytoplasm of almost all cells in SNpc (data not shown). N-SYN was also effectively directed to the intracellular space of DA neurons, indicating that nitration also does not affect internalization *in vivo* (Figure 7). As shown in Figure 8, a single injection of the positive control 6-OHDA (2 µl of 8 µg/µL) severely decreased the number of TH-positive neurons in the injected SNpc relative to the internal control SNpc 5 weeks and 11 weeks after the injection (67.6±1.5% less at 5 weeks; 69.3±3.0% less at 11 weeks; Figure 8A and B), demonstrating the efficacy of our delivery method. In contrast, injecting 2 µL of N-GFP (20 µmol/L) every day for 2 weeks did not significantly affect the population of DA neurons in the SNpc 5 weeks or 11 weeks later (8.0±4.0% reduction at 5 weeks; 11.0±5.1% reduction at 11 weeks), thus showing the specificity of the lesion (Figure 8A and B). Relative to the N-GFP treatment, daily infusions of SYN (2 µL of 20 µmol/L) for 2 weeks also did not have a significant effect on DA neuron survival 5 weeks or 11 weeks later (13.4±5.4% reduction at 5 weeks; 14.5±7.7% reduction at 11 weeks; Figure 8A and B). Interestingly, 5 weeks after administration of N-SYN (2 µL of 20 µmol/L), the number of TH-positive neurons in the SNpc was reduced by a third (34.5±3.0%; Figure 8A, B and C), and 11 weeks after treatment the number was reduced even further (48.7±4.1%; Figure 8A, B and D). These results demonstrate that acute exposure to nitrated α-synuclein causes a long-lasting and progressively more severe cytotoxic effect on DA neurons in the SNpc of adult rats.

To ensure that loss of TH immunoreactivity was due to neuronal loss and not a simply abandoning the function of dopamine production, and to assess any potential toxicity on the contralateral side (possibly due to inflammation), Nissl-positive and TH-positive neurons were quantified on both sides in N-SYN group (Figure 8E). Correlation analysis of total Nissl-positive neurons compared to TH-positive and TH-negative neurons demonstrated that the number of total neurons correlated with numbers of TH-positive neurons compared to numbers of TH-negative neurons (r: 0.825 *vs.* 0.121 at 5 weeks; r: 0.915 *vs.* 0.660 at 11 weeks). This confirmed that differences in TH-positive neuron counts were due to differences in numbers of structurally intact neurons and eliminated the possibility that differences resulted from the down-regulation of TH itself. Also, quantification of dopaminergic neurons on both ipsi- and contralateral sides were not reminiscent of remarkable toxicity on the contralateral side SNpc of rats in N-SYN group.

Further more, we found the GABAergic neurons of the substantia nigra pars reticulata (SNpr) (Figure S2) and DA neurons of VTA (data not shown) were spared in rat microinjected with N-SYN for two weeks. These data support that SNpc DA neurons are indeed more sensitive to N-SYN than the neighboring GABAergic neurons in the SNpr or the DA neurons of VTA. Immunostaining applying anti-HA antibody (Figure 7) ruled out the possibility that the N-SYN did not reach the SNpr.

**Figure 9:**
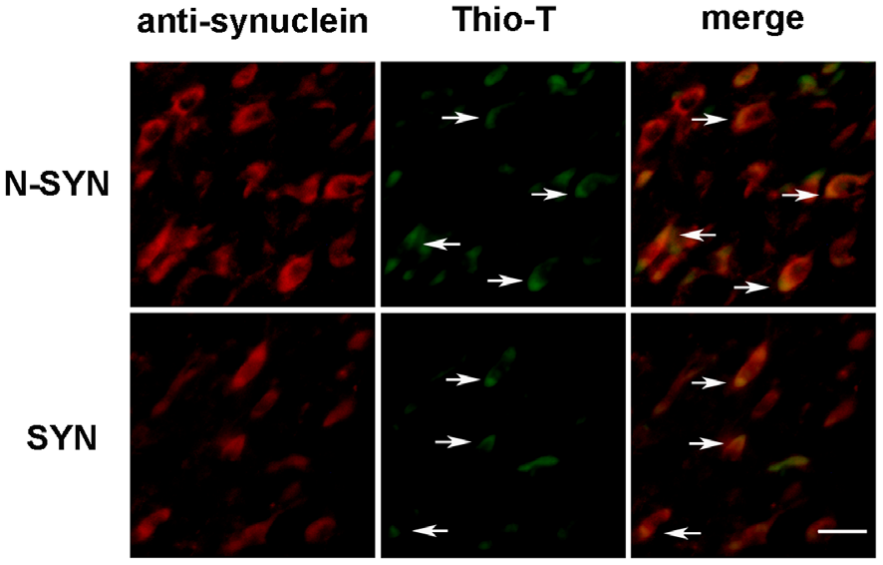
N-SYN and SYN can form intracellular aggregates in the substantial nigra. Anti-α-synuclein antibody (red) and Thioflavin-T (green) were applied to detect aggregation after N-SYN or SYN treatment. α-Synuclein positive intracellular aggregates, which were also Thioflavin-T positive, were formed after two weeks injection of N-SYN (arrows in upper panel). Similar aggregates were detected after two weeks injection of SYN (arrows in lower panel). Scale bar: 25 µm.

We also detected whether the fusion proteins, SYN and N-SYN, could form intracellular aggregates in the substantial nigra. Consistent with former reports [32], SYN infusion induced aggregation intracellularly (Figure 9, arrows in lower panel). Interestingly, we also found N-SYN infusion could induce α-synuclein and Thioflavin-T positive intracellular aggregates (Figure 9, arrows in upper panel).

### Microglia and astrocyte were activated in the substantia nigra of rat 5 weeks after N-SYN injection

**Figure 10:**
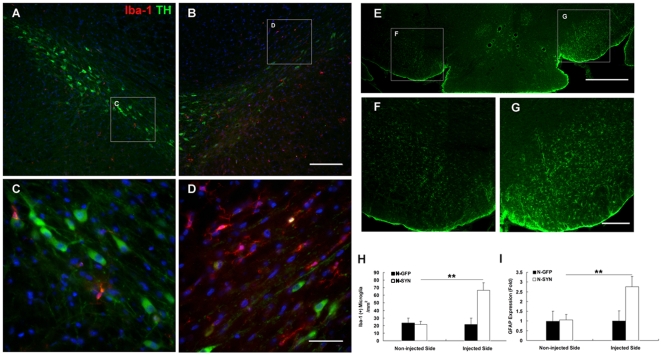
Increased microglia and astrocyte activation in the substantia nigra of rat in response to N-SYN. Coronal VM sections of N-SYN group rats showed loss of DA neurons (TH, green) concurring with mounting microglia (Iba-1, red) in substantia nigra of ipsilateral side (B) *vs*. that in substantia nigra of contralateral side (A). (C) and (D) representing a magnification of boxed area in (A) and (B), respectively. More microglia exhibited activated morphology were found close to atypical DA neurons on the N-SYN-injected side (B, D). Quantifications showed there were more Iba-1-positive microglia on the N-SYN-injected side (66.5±9.9/mm^2^) *vs*. those on the non-injected contralateral side (21.5±3.8/mm^2^, *p*<0.01) (H). Scale bars: (A, B), 200 µm; (C, D), 50 µm. Coronal VM sections of N-SYN group rats showed astrocyte activation (GFAP, green) in SNpc of ipsilateral side (E). (F) and (G) representing a magnification of boxed area in (E). Quantification of anti-GFAP immunostaining showed the level of GFAP expression in N-SYN group was augmented to 2.7±0.5 fold on the ipsilateral side *vs*. that on the contralateral side (*p*<0.01) (I). Scale bars: (E), 800 µm; (F, G), 200 µm. Data represent mean ± SEM from three independent experiments. **, *p*<0.01.

Microglia and astrocyte were activated in the substantia nigra of rat 5 weeks after N-SYN injection (Figure 10). Coronal VM sections of N-SYN group rats showed loss of DA neurons concurring with mounting microglia in substantia nigra of ipsilateral side *vs*. that in substantia nigra of contralateral side. More microglia exhibited activated morphology were found close to atypical DA neurons on the N-SYN-injected side (Figure 10B, D). Quantifications showed there were more Iba-1-positive microglia on the N-SYN-injected side (66.5±9.9/mm^2^) *vs*. those on the non-injected contralateral side (21.5±3.8/mm^2^, *p*<0.01) (Figure 10H). Anti-GFAP immunostaining also showed the level of GFAP expression in N-SYN group was augmented to 2.7±0.5 fold on the ipsilateral side *vs*. that on the contralateral side (*p*<0.01) (Figure 10I). Thus, microglia and astrocyte were activated in the substantia nigra of rat 5 weeks after N-SYN injection.

### N-SYN-treated rats show reduced locomotion in the open field test

**Figure 11:**
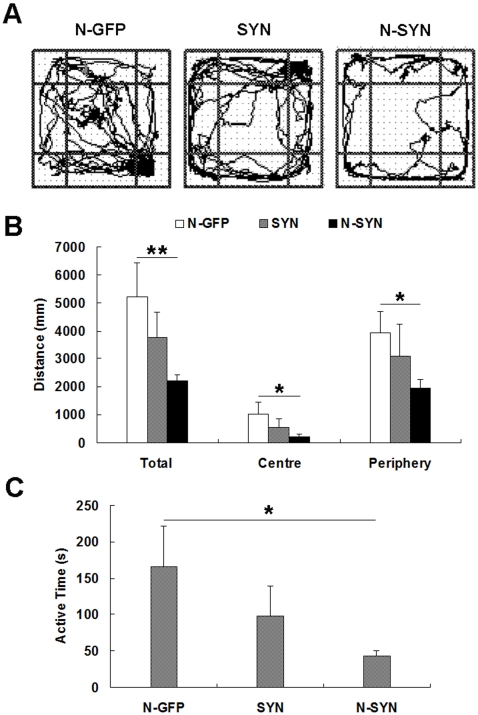
Reduced locomotor activity of N-SYN-treated rats during the open field test. (A) Representative locomotor activity paths from N-SYN, SYN and N-GFP groups are shown. (B) Rats from the N-SYN group exhibited reduced locomotor activity compared with those in the N-GFP group. Total distance, distance traveled in centre, and distance traveled in the periphery for the N-SYN group were all significantly less than those of the N-GFP group. (C) Active time for rats in the N-SYN group was less than that in the N-GFP group. Values represent mean ± SEM; *, *p*<0.05; **, *p*<0.01. n = 8 per group.

To determine the effect of N-SYN on locomotor and exploratory behavior, we evaluated the performance of N-SYN- and N-GFP-treated rats in an open field test (Figure 11A). N-GFP, SYN or N-SYN (20 µmol/L) were injected unilaterally into the SNpc daily for 2 weeks and then total distance traveled, distance traveled in the center and distance traveled in the periphery during a 15 min time period were measured. Compared to the N-GFP-treated group, infusing N-SYN caused significant decreases of more than 50% in all three parameters (*p*<0.05, Figure 11B). Treating with SYN also significantly reduced locomotion and exploratory behavior compared to the N-GFP group (*p*<0.05, Figure 11B), but the reductions were significantly less severe than in the N-SYN group. Overall active time was concomitantly reduced in the N-SYN group, and to a significantly greater degree than the SYN-treated group (Figure 11C). To exclude the possibility that the reduction in distance traveled was the result of slow movements, we measured the average velocity by dividing total distance traveled by the active time. No significant decline in velocity was observed in the N-SYN group (52.0±5.9 mm/s, *p*>0.05). To determine whether N-SYN treatment affected the exploratory behavior, we calculated the distance traveled in the centre or periphery as a percentage of total distance traveled. No statistical difference was detected (data not shown). Together, these data suggest that N-SYN-treated rats, by consistently failing to initiate voluntary movement, exhibit reduced locomotion, which is a feature of PD [38]. This impairment in mobility is possibly due to compromised nigrostriatal functioning.

### N-SYN-treated rats show impaired motor coordination in the rotorod test

**Figure 12:**
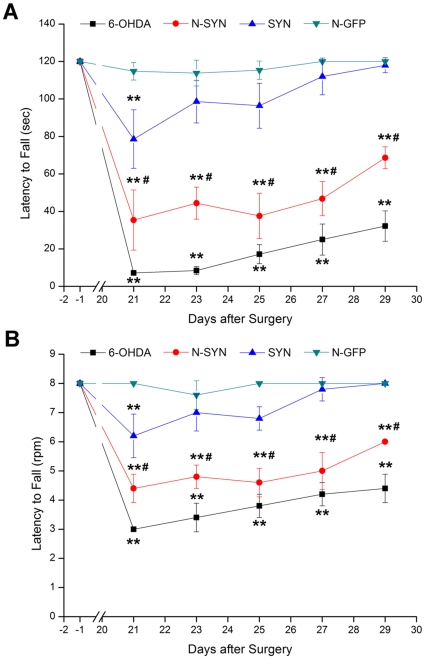
Impairment of motor coordination in N-SYN-treated rats during the rotorod test. The motor defects caused by N-SYN treatment were further assessed by comparing the latency to fall from the rotorod between rats from all four treatment groups (6-OHDA, N-SYN, SYN, and N-GFP) at each indicated time point, before and after surgery. Latency to fall off the rotating rod was significantly reduced in the N-SYN group relative to N-GFP or SYN groups at all time points. Values represent mean ± SEM; **, *p*<0.01 *vs.* N-GFP; #, *p*<0.01 *vs.* SYN. n = 8 per group.

Gait disturbance and postural instability are also major motor manifestations of PD [38]. We thus asked whether unilaterally infusing N-SYN into the SNpc everyday for two weeks causes motor coordination impairment. To evaluate neurotoxicity in a nitrated α-synuclein lesion model *in vivo*, coordination and balance was evaluated for the N-SYN group using the rotorod test. Latency to fall from the rod, assessed as a function of both time (Figure 12A) and rotational velocity (Figure 12B), was significantly reduced in the N-SYN group compared to the N-GFP or SYN groups at the time points throughout the entire span of the experiment (days 21 to 29 post-surgery; *p*<0.01). These results demonstrate that N-SYN impairs motor coordination and balance.

### N-SYN rats display motor asymmetry during the rotation test

**Figure 13:**
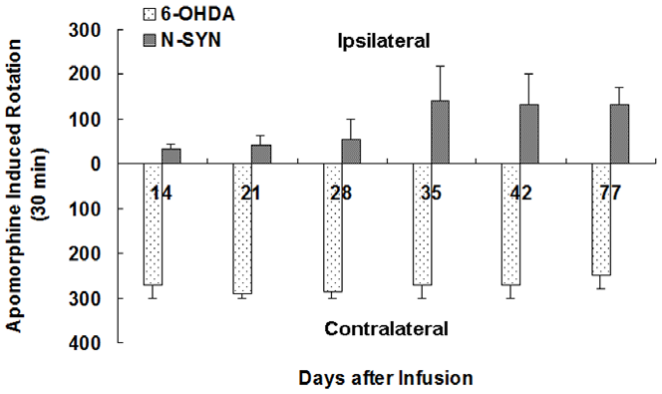
Rats treated with N-SYN display motor asymmetry induced by apomorphine. Motor asymmetry was assessed by administrating apomorphine (APO; 0.5 mg/kg) at the indicated time point after 6-OHDA or N-SYN infusion. Contrarily to 6-OHDA treatment, which caused contralateral rotation, rats in the N-SYN group showed continuous ipsilateral rotation. Values represent mean ± SEM. n = 8–11 per group.

Both N-SYN- and 6-OHDA-injected rats presented apomorphine (APO)-induced continuous unilateral rotational movement two weeks after treatment (Figure 13A). Surprisingly, and contrary to 6-OHDA which caused contralateral rotation, N-SYN treatment caused continuous net ipsilateral rotation (the total number of rotations in the ipsilateral or contralateral direction) at all time points (Figure 13B), although a subset of rats were more tolerant to N-SYN than the majority. This type of behavior is suggests irreversible unilateral damage to the nigrostriatal DAergic pathway, as has been described previously. No unilateral continuous rotation was detected in rats treated with equivalent doses of SYN for comparable time periods (data not shown). Thus, the nitrated form α-synuclein is deleterious to the nigrostriatal system.

### Dopamine D2 receptor is down-regulated and striatal DA and DOPAC level is depleted by N-SYN

**Figure 14:**
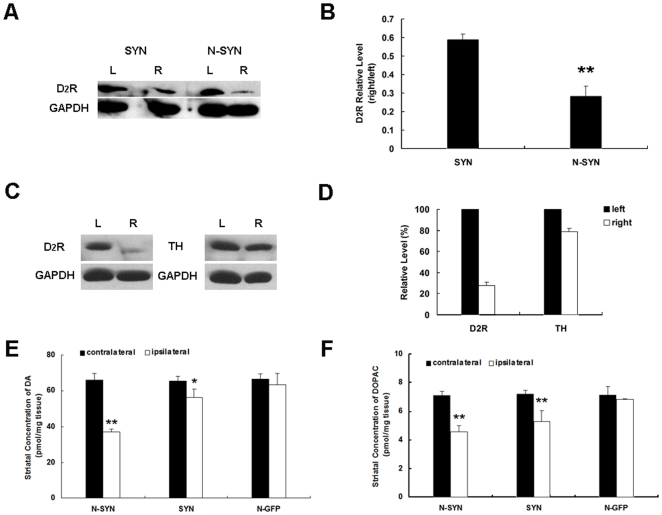
Dopamine D2 receptor is downregulated and striatal DA and DOPAC level is depleted by N-SYN. (A) Western blot showing dopamine D2 receptor (D_2_R) protein levels in the striatum of N-SYN- or SYN-treated rats five weeks postsurgery. GAPDH was used as a loading control. (B) Graph showing the relative levels of D_2_R (injected side *vs.* intact side) in the striatum of N-SYN- and SYN-treated rats. N-SYN and SYN treatment induced the down-regulation of D_2_R to 26.1±4.7% and 59.5±3.3% of control, respectively. (C) Further comparison of Tyrosine hydroxylase (TH) and D_2_R protein levels in bilateral striatum of N-SYN-treated rats by Western blot. (D) Graph showing the relative levels of D_2_R and TH (injected side *vs.* intact side) in the striatum of N-SYN-treated rats. N-SYN treatment induced the down-regulation of TH to 78.9±3.2% of control. Values represent mean ± SEM. ** *p*<0.01, n = 4 per group. L, left; R, right. The amounts of DA and DOPAC in striatal tissue were measured on the injected and uninjected sides of individual animals in N-SYN, SYN and control (N-GFP) as described in Materials and Methods and were displayed as the average striatal concentration of DA (E) and DOPAC (F) on both ipsi- and contralateral sides. Values represent mean ± SEM. * *p*<0.05, ** *p*<0.01, n = 3 per group.

APO-induced contralateral rotation behavior seen in 6-OHDA-lesioned rats is primarily mediated by the postsynaptic dopamine D2 receptor (D_2_R) [39], [40]. To investigate the mechanism underlying APO-induced rotation in rats treated with N-SYN, we first determined the levels of D_2_R on both the injected and intact sides of the striatum by immunoblotting 5 weeks post surgery. In the N-SYN group, levels of D_2_R were significantly lower on the treated side (right) of the striatum than that on the intact side (left; Figure 14A). Quantification of band intensity showed that D_2_R levels on the injected side were reduced to 26.1±4.7% of the intact side (Figure 14B). For D_2_R is present both in pre- and post-synaptic populations in the striatum, levels of TH, a presynaptic markers of nigrostriatal DA neurons, were further detected. TH levels of the treated side were reduced to 78.9±3.2% of the intact side (Figure 14C, D). Considering the discrepancy of reduced levels between D_2_R and TH in striatum of N-SYN group, we supposed that the decrease of D_2_R may be not only attributed to down-regulation of presynaptic D_2_R but also to down-regulation of post-synaptic D_2_R. Additionally, a milder decrease in D_2_R levels was observed with SYN treatment (injected side reduced to 59.5±3.3% of intact side; Figure 14A and B).

To further investigate the mechanisms underlying APO-induced rotation, the lesion on the nigrostriatal DAergic pathway at the striatal level was detected by measuring the striatal DA and DOPAC level. N-SYN group displayed severe reduction in DA and DOPAC compared with control (N-GFP group). At 5 weeks post surgery, in N-SYN group, compared with the non-injected side, level of DA and DOPAC on the injected side was depleted by 55.9±5.2% (*p*<0.01 compared with N-GFP) and 64.4±3.3% (*p*<0.01 compared with N-GFP) respectively, while in SYN group, level of DA and DOPAC was depleted by 86.0±4.7% (*p*<0.05 compared with N-GFP) and 81.2±2.8% (*p*<0.01 compared with N-GFP) respectively (Figure 14E, F). Thus, N-SYN induced more severe depletion of DA and DOPAC compared with SYN treatment, which mirrored the loss of DA neurons at the same time point (Figure 8B).

## Discussion

Environmental factors, mitochondrial dysfunction, misfolded protein aggregates, ubiquitin-proteasome system impairment, and oxidative stress have all been implicated in the degeneration of DA neurons that occurs in PD [41]. A number of nitrated proteins have found to be associated with the pathogenesis of PD, including protein kinase C [20], catecholamine [21], manganese superoxide dismutase [22], α-synuclein [23], [24], [25], and others. However, no direct evidence has shown whether and which nitrated protein(s) is/are toxic to DAergic cells, thus contributing to the onset and progression of PD. In the present study, we synthesized recombinant α-synuclein in which all three tyrosine residues were nitrated (N-SYN), consistent with the form of the protein that has been identified in postmortem brain tissue of patients with PD [23]. Using LDH and TUNEL assays, we demonstrated that N-SYN was more cytotoxic to SHSY-5Y cells and DA neurons, both *in vivo* and *in vitro*, than unnitrated SYN. Continual unilateral infusion of N-SYN into the SNpc of rats caused chronic motor deficits, such as low level of spontaneous activity, poor coordination and asymmetry, that were significantly more severe than unnitrated SYN but milder than those caused by injecting 6-OHDA into the SNpc. The neurotoxic effects of N-SYN on DA neurons of the SNpc, combined with behavior deficits that we observed, strongly suggest that is sufficient to cause a PD-like disorder in rats. Thus, our study provides the first evidence that nitrated α-synuclein can induce the death of DA neurons.

Although α-synuclein is ubiquitously expressed and Lewy pathology has been detected throughout the CNS and peripheral autonomic nervous system, compared with other cell types, DA neurons in the substantia nigra are highly susceptible in PD associated pathology. As an example of this selectivity, transfecting human α-synuclein into the substantia nigra of rats selectively damages the nigrostriatal DA system, despite high levels of transgene expression in non-DA neurons [42]. As DA metabolism generates high concentrations of reactive oxygen species/reactive nitrogen species (ROS/RNS) [43], [44], we suggest that the interaction between α-synuclein and ROS/RNS in DA neurons contributes to selective nigrostriatal neurodegeneration in PD. In the presence of metal, α-synuclein has been found to become even more toxic to DAergic cells, likely due to increased rates of ROS/RNS formation [43], [45]. On the other hand, α-synuclein-deficient mice are resistant to toxicity induced by MPTP and other mitochondrial toxins [46], [47]. Moreover, α-synuclein in its fully nitrated form (i.e. nitrated at all three of its tyrosine residues) is considered a marker for oxidative and nitrative stress, and has been found to accumulate in the filamentous building blocks of Lewy bodies in PD [23]. These findings and others, combined with the results presented herein, strongly suggest that both RNS and α-synuclein are major factors in the pathogenesis of PD. What then, might render nitrated α-synuclein more toxic to DA neurons? Although the mechanism remains unclear, several possibilities have been proposed. These include: (1) nitration inhibits fibrillation of human α-synuclein *in vitro* by forming stable soluble oligomers or protofibrils [24], [48], [49]; (2) nitration reduces the association of α-synuclein with lipid vesicles, prolonging its intracellular half-life [50], thus increasing cytosolic concentrations that possibly cause and exacerbate ER-Golgi traffic blocks [51]; and (3) nitrated α-synuclein may contribute to the formation of annular protofibrils that resemble a class of pore-forming bacterial toxins [52].

Nitrated tyrosine (NT) modifications in α-synuclein do not seem to occur merely at the very end stage of PD. Our *in vitro* and *in vivo* observations support the idea that nitrated α-synuclein itself directly contributes to the DAergic neurodegeneration that occurs during the progression of PD. Recent studies [25], [53] have shown that increased nitration of tyrosine residues in α-synuclein induce immune responses that intensify PD progression. In the present study, we found that intracellular nitrated α-synuclein can cause a rapid DAergic cell apoptosis response *in vitro*, independent of an immune response. Although this result does not rule out the role for N-SYN in the activation of microglia or T cell immunoreactivity, this finding suggests nitrated α-synuclein is sufficient to kill DA neurons in a cell autonomous manner. Thereupon, nitrated α-synuclein inclusions would be phagocytosed by microglia or presented to T cell lymphocytes [25], [53], [54] causing neuroinflammation and immune responses which may induce additional α-synuclein nitration due to a burst in the production of free radicals [55], [56]. This would in turn aggravate DA neuron loss.

In this study, we didn't observe remarkable toxicity with SYN infusion at the level of SNpc, which was not consistent with Kirik's study using adeno-associated viral vectors [12]. It could be attributed to several factors, including: (1) whether the α-synuclein is adequately delivered to substantia nigra; (2) whether DA neuron is more sensitive to intracellular expressed α-synuclein mediated by viral infection. There are four transcript variants of α-synuclein described so far. Among these variants, SNCA112, with a shortening of the C-terminal, is surmised to have increased propensity to aggregate and is over-expressed in dementia with Lewy bodies compared with full-length α-synuclein [57]. Considering the absence of a site important for the inhibition of α-synuclein fibril formation, SNCA112 may increase its propensity to fibrillize into a less noxious form [58]. Thus α-synuclein tested in this study may have less toxicity compared with α-synuclein used in Kirik's study.

Though less toxic than N-SYN, the SYN in itself is toxic, and induces nigrastriatal degeneration when overloaded. It can be attributed to several factors, including: (1) SYN may inhibit proteasomal function and induces protein degradation failure when overloaded intracellularly; (2) SYN can be modified into more toxic forms by peroxynitrite, reactive oxygen species and/or other endogenous oxidative compounds, and aggravates nigrostriatal degeneration.

Administration of 6-OHDA to the SNpc is a routine procedure for generating rodent Parkinsonian models [59]. There are several shortcomings to the 6-OHDA model of PD, however, including the unnaturally acute damage that 6-OHDA causes, and the lack of evidence that 6-OHDA is detected in DA neurons in the pathogenesis of PD. In this study, N-SYN emerged as a novel candidate with which to develop a new molecular model for PD. Using N-SYN to model PD pathogenesis offers two major advantages: N-SYN is found in ventral mesencephalic DA neurons of PD patients and is considered a critical factor in PD development [23], [25], thus providing a pathophysiological basis for this model; and, as we showed herein, nitrated α-synuclein affects DA neuron less severely and causes less acute lesions than 6-OHDA *in vivo*. Thus, the N-SYN administration paradigm we used herein, which caused a slower and milder, and thus more physiologically relevant, development of Parkinsonian lesions and behaviors, validates its use as a potentially *in vivo* model of PD, particularly on studying the toxicity of PD associated protein modification.

The results of our rotation tests revealed that ipsilateral administration of N-SYN to the SNpc of adult rats caused the animals to continuously rotate ipsilateral to the lesioned side after apomorphine induction, and a concomitant ipsilateral down-regulation of post-synaptic D_2_R within the striatum. It has previously been shown that TH-positive nerve terminals within the striatum are reduced in transgenic mice overexpressing α-synuclein [13], and D_2_R is downregulated in some neurodegenerative diseases [60]. Recently, de Oliveira et al. reported that D_2_R down-regulation occurred together with the upregulation of 3-nitrotyrosine and α-synuclein in a rodent oxidative stress model [61], although no cause-effect relationship was ascertained. In this study, we demonstrated that N-SYN not only led to the death of DA neurons in the SNpc, but also to a down-regulation of D_2_R in the striatum. We hypothesize that nitrated α-synuclein triggered DA neuron dystrophy in the SNpc, leading to the retraction of the spines and dispersal of post-synaptic machinery (including D_2_R receptors) on dendrites of downstream striatal projection neurons [62]. Considering behavioral and histological data in our study showing N-SYN caused milder lesion to DA neurons in the SNpc than 6-OHDA and the data from HPLC indicating striatal dopamine depletion in N-SYN-treated rats, another possible explanation may be that as described previously [63]: The difference in rotation behavior was caused by differences in the magnitude of the nigrostriatal lesion. It was the milder lesion of DA neurons in the SNpc that depleted striatal dopamine, reduced the binding of D1 receptors in the ipsilateral striatum and subsequently caused apomorphine induced ipsilateral rotation. Further study is needed to elucidate the exact role of dopamine receptors, including D_1_R and D_2_R, in the rotation behavior.

In summary, we here provide evidence to show that α-synuclein exclusively in its nitrated form is sufficient to cause the death of SNpc DA neurons in a cell autonomous manner *in vitro*, and to cause lesions in the SNpc and behavior deficits that resemble those suffered by patients with PD. This study opens new avenues to study the role of specific protein modification in neurodegenerative disease.

## Supporting Information

Figure S1 N-SYN induces protein aggregation and apoptotic cell death of SHSY-5Y cells. Immunostaining with anti-HA antibody (red) and TUNEL (green) revealed that some TUNEL (+) apoptotic cells contained intracellular aggregates (arrow), but some TUNEL (+) apoptotic cells didn't contain aggregates (arrow head). Additionally, some cells contained HA (+) aggregates with TUNEL (−) (asterisk). Scale bar: 10 µm.
(1.40 MB TIF)

Figure S2 GABAergic neurons in the substantia nigra reticular (SNpr) were spared in N-SYN treated rats 11 weeks post injection. TH (green, dopaminergic) and glutamic acid decarboxylase (GAD) (red, GABAergic) immunostaining of SN showed loss of dopaminergic but not of GABAergic neurons after microinjections with N-SYN (right side) for two weeks. Scale bars: (A), 500 µm; (B, C), 200 µm; (D, E), 100 µm.
(4.82 MB TIF)
