# Supplementary figures and images for: Nitrated α-Synuclein Induces the Loss of Dopaminergic Neurons in the Substantia Nigra of Rats
Source: PLoS One. 2010 Apr 8;5(4):e9956. doi: 10.1371/journal.pone.0009956 (PMC2851648; doi:10.1371/journal.pone.0009956)

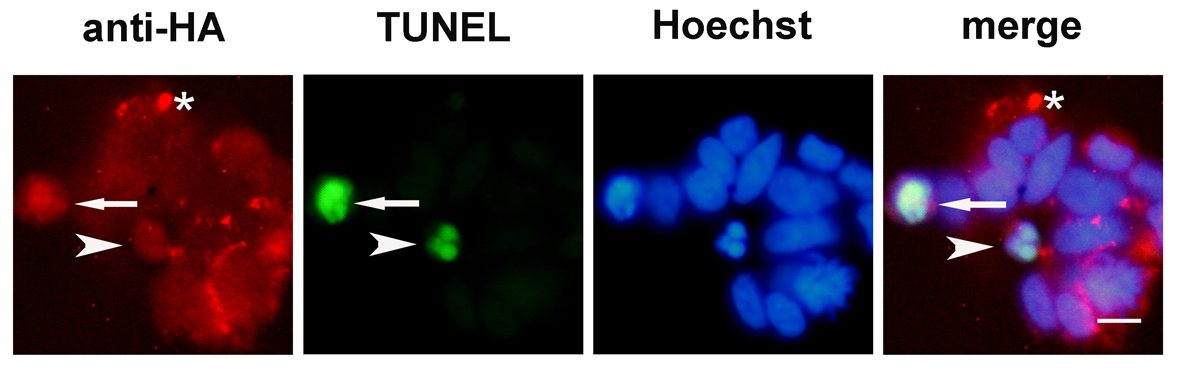

Supplement: Figure S1 — N-SYN induces protein aggregation and apoptotic cell death of SHSY-5Y cells. Immunostaining with anti-HA antibody (red) and TUNEL (green) revealed that some TUNEL (+) apoptotic cells contained intracellular aggregates (arrow), but some TUNEL (+) apoptotic cells didn't contain aggregates (arrow head). Additionally, some cells contained HA (+) aggregates with TUNEL (−) (asterisk). Scale bar: 10 µm. (1.40 MB TIF) [file pone.0009956.s001.tif]

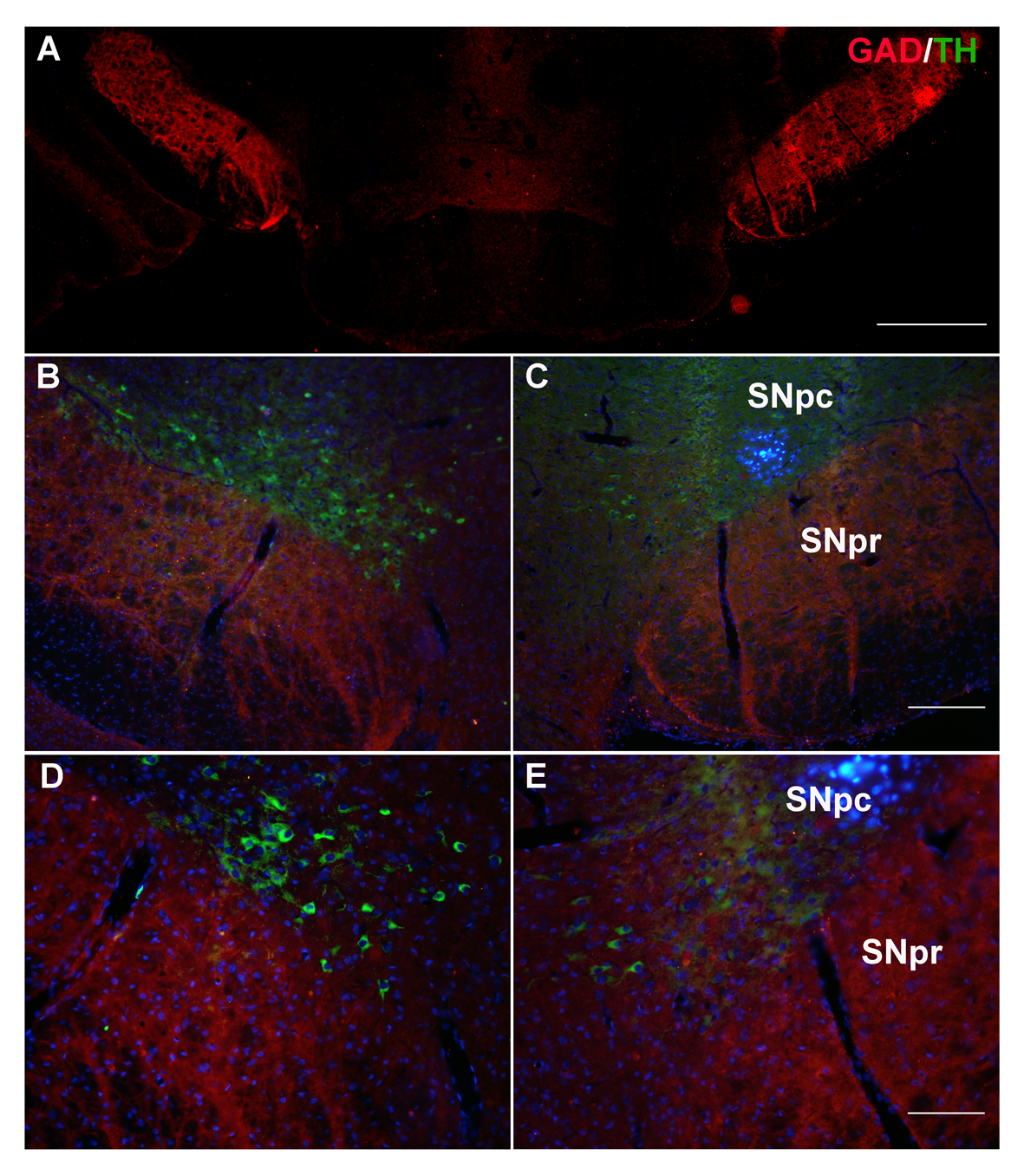

Supplement: Figure S2 — GABAergic neurons in the substantia nigra reticular (SNpr) were spared in N-SYN treated rats 11 weeks post injection. TH (green, dopaminergic) and glutamic acid decarboxylase (GAD) (red, GABAergic) immunostaining of SN showed loss of dopaminergic but not of GABAergic neurons after microinjections with N-SYN (right side) for two weeks. Scale bars: (A), 500 µm; (B, C), 200 µm; (D, E), 100 µm. (4.82 MB TIF) [file pone.0009956.s002.tif]
